# Linkages to HIV confirmatory testing and antiretroviral therapy after online, supervised, HIV self‐testing among Thai men who have sex with men and transgender women

**DOI:** 10.1002/jia2.25448

**Published:** 2020-01-20

**Authors:** Nittaya Phanuphak, Jureeporn Jantarapakde, Linrada Himmad, Thanthip Sungsing, Ratchadaporn Meksena, Sangusa Phomthong, Petchfa Phoseeta, Sumitr Tongmuang, Pravit Mingkwanrungruang, Dusita Meekrua, Supachai Sukthongsa, Somporn Hongwiangchan, Nutchanin Upanun, Supunnee Jirajariyavej, Tanate Jadwattanakul, Supphadith Barisri, Tippawan Pankam, Praphan Phanuphak

**Affiliations:** ^1^ PREVENTION Thai Red Cross AIDS Research Centre Bangkok Thailand; ^2^ Service Workers IN Group (SWING) Foundation Bangkok Thailand; ^3^ SWING Foundation Pattaya Thailand; ^4^ Rainbow Sky Association of Thailand Bangkok Thailand; ^5^ Sisters Foundation Pattaya Thailand; ^6^ Taksin Hospital Bangkok Thailand; ^7^ Queen Savang Vadhana Memorial Hospital Pattaya Thailand; ^8^ Anonymous Clinic Laboratory Thai Red Cross AIDS Research Centre Bangkok Thailand

**Keywords:** online, HIV self‐testing, link to care, men who have sex with men, transgender women

## Abstract

**Introduction:**

Online, supervised, HIV self‐testing has potential to reach men who have sex with men (MSM) and transgender women (TGW) who never tested before and who had high HIV‐positive yield. We studied linkages to HIV confirmatory test and antiretroviral therapy (ART) initiation among Thai MSM and TGW who chose online and/or offline platforms for HIV testing and factors associated with unsuccessful linkages.

**Methods:**

MSM and TGW were enrolled from Bangkok Metropolitan Region and Pattaya during December 2015 to June 2017 and followed for 12 months. Participants could choose between: 1) offline HIV counselling and testing (Offline group), 2) online pre‐test counselling and offline HIV testing (Mixed group) and 3) online counselling and online, supervised, HIV self‐testing (Online group). Sociodemographic data, risk behaviour and social network use characteristics were collected by self‐administered questionnaires. Linkages to HIV confirmatory testing and/or ART initiation were collected from participants who tested reactive/positive at baseline and during study follow‐up. Modified Poisson regression models identified covariates for poor retention and unsuccessful ART initiation.

**Results:**

Of 465 MSM and 99 TGW, 200 self‐selected the Offline group, 156 the Mixed group and 208 the Online group. The Online group demonstrated highest HIV prevalence (15.0% vs. 13.0% vs. 3.4%) and high HIV incidence (5.1 vs. 8.3 vs. 3.2 per 100 person‐years), compared to the Offline and Mixed groups. Among 60 baseline HIV positive and 18 seroconversion participants, successful ART initiation in the Online group (52.8%) was lower than the Offline (84.8%) and Mixed groups (77.8%). Factors associated with unsuccessful ART initiation included choosing to be in the Online group (aRR 3.94, 95% CI 1.07 to 14.52), <17 years old at first sex (aRR 3.02, 95% CI 1.15 to 7.92), amphetamine‐type stimulants use in the past six months (aRR 3.6, 95% CI 1.22 to 10.64) and no/single sex partner (aRR 3.84, 95%CI 1.36 to 10.83) in the past six months.

**Conclusions:**

Online, supervised, HIV self‐testing allowed more MSM and TGW to know their HIV status. However, linkages to confirmatory test and ART initiation once tested HIV‐reactive are key challenges. Alternative options to bring HIV test confirmation, prevention and ART services to these individuals after HIV self‐testing are needed.

## 1 Introduction

HIV testing uptake among key populations (KPs), in particularly men who have sex with men (MSM) and transgender women (TGW) in Thailand, has been low over the past decade 1. Use of HIV self‐testing is one additional option to increase access to HIV testing among individuals with barriers for conventional HIV testing. HIV self‐testing has been shown to engage high proportion of first‐time testers with high HIV‐positive yield 2.

Successful linkage rates to HIV confirmatory testing and antiretroviral therapy (ART) among MSM who tested reactive by HIV self‐testing were reported in a range of 31% to 100% from studies conducted in resource‐limited and high‐income countries 3, 4, 5. Factors associated with unsuccessful linkages to HIV confirmatory testing and/or ART, however, have not been extensively explored due to a survey nature of studies and/or small number of MSM participants who tested reactive by HIV self‐testing.

In the Asia‐Pacific region, the overall ART coverage among people who know their HIV‐positive status remained sub‐optimal 6. Data on ART coverage among KPs have been limited and likely to be lower than the national figures due to common structural barriers 7. Although HIV self‐testing has high potential to increase the number of people who know their HIV‐positive status, unsuccessful linkages of those who tested reactive to HIV confirmatory testing and ART initiation would make HIV programmes futile. It would be impractical to scale‐up HIV self‐testing without having a plan in place on how to immediately link people to ART and HIV prevention programmes.

In this paper, we reported linkages to HIV confirmatory testing and ART initiation among Thai MSM and TGW who chose to have online, supervised HIV self‐testing and tested reactive, compared to those who tested positive through offline HIV testing strategies. We also studied factors associated with unsuccessful linkage to ART initiation among these populations.

## 2 Methods

We consecutively recruited and enrolled Thai MSM and TGW, during December 2015 to June 2017, into a 12‐month cohort study with 6‐monthly visits to assess the preferences and feasibility of online and offline HIV counselling and testing strategies (NCT03203265). This study was approved by the Institutional Review Board of the Faculty of Medicine, Chulalongkorn University and the Bangkok Metropolitan Administration Ethics Committee. MSM and TGW were eligible for the study if they were Thai national, aged >18 years, engaged in unprotected anal sex with men at least once in the past six months, living in Bangkok Metropolitan Region or Pattaya, and not known to be HIV positive.

The study was conducted by the Thai Red Cross AIDS Research Centre (TRCARC), Service Workers IN Group (SWING) Foundation, Rainbow Sky Association of Thailand (RSAT) and Sisters Foundation. SWING, RSAT and Sisters were community‐based organizations (CBOs) who have provided HIV‐related services to MSM and TGW since 2015, with capacity building support from the United States Agency for International Development (USAID)‐funded LINKAGES Thailand Project, as part of the Key Population‐Led Health Services (KPLHS) model to address service gaps for KPs in Thailand 8. Through KPLHS, defined sets of HIV‐related health services were designed and delivered by trained lay providers who were members of KP communities, in close partnerships with public health sectors.

The study was supported by amfAR GMT Initiative grant. Factors associated with preferences for online or offline HIV services at study entry were previously reported 9. Here we reported findings on seroconversion, retention and linkages to HIV confirmatory testing and ART initiation during the 12‐month study period.

### 2.1 Study recruitment

Study posters, messages and an online HIV self‐testing video were promoted through CBOs’ websites and platforms commonly used by MSM and TGW such as Facebook, LINE (the most popular instant communications app used in Thailand), Camfrog video chat rooms and Hornet for online study recruitment. CBO staff also conducted offline recruitment at hot spots using study posters and flyers. Individuals interested in joining the study were subsequently scheduled for an informed consent process.

### 2.2 HIV counselling and testing via online and/or offline strategies

Enrolled participants were allowed to self‐select from three HIV counselling and testing strategies including: (1) offline HIV counselling and testing (Offline group), (2) online pre‐test counselling and offline HIV testing (Mixed group) and 3) online counselling and online supervised HIV self‐testing (Online group).

All HIV testing algorithms were conducted according to Thailand National Guidelines 10. In the Mixed group, pre‐test counselling was conducted online and participants were scheduled to receive HIV testing and post‐test counselling at a by‐appointment‐only clinic in Bangkok. In the Online group, participants received online pre‐test counselling and had an HIV self‐testing package and an online HIV self‐testing video sent to them. Study staff contacted each participant to ensure package delivery and schedule time for the online, supervised, HIV self‐testing process. Step‐by‐step, real‐time guidance was provided through a video chatting platform preferred by each participant. For self‐testing, we used a rapid 3^rd^ generation assay (Alere Determine^TM^ HIV‐1/2, Alere Medical Co., Ltd., Matsuhidal, Matsudo‐shi, Chiba, Japan) and fingerprick blood sample.

### 2.3 Other services offered

Symptom screening for other sexually transmitted infection (STI) testing was conducted during the counselling sessions. Laboratory STI test was ordered (in the Offline group) or participants were referred to laboratory STI test (in the other groups), based on provider’s judgement. Pre‐exposure prophylaxis (PrEP) and post‐exposure prophylaxis (PEP) were offered to all eligible participants and assistance to access offline services was given.

### 2.4 Referral of HIV‐positive participants to treatment facilities

ART could be initiated regardless of CD4 count in Thailand since 2014 as per National Guidelines 10. All Thai citizens could access ART and related laboratory services without charge through National Health Security Office, Social Security Office, or Civil Servant health insurance schemes. Transfer of health insurance from an automatically/previously registered hospital to a preferred hospital, for logistical or confidentiality reasons, could take two weeks to a year. HIV‐positive participants in all groups received support from study staff to identify their preferred hospitals and were encouraged to visit these hospitals for HIV confirmatory testing and/or ART initiation as soon as possible after HIV testing. Participants in the Offline and Mixed groups had a referral letter, along with a report of confirmatory HIV test results, issued to them to assist the referral. Participants in all groups could request to have study staff accompanying them to the referred hospitals. Study staff contacted referred participants at week 2, months 1, 3, 6 and 12 in the Offline and Mixed groups, and at day 3, weeks 1, 2, and every two to four weeks in the Online group, to ensure successful referral and/or offer additional support as needed.

### 2.5 Study follow‐up visits

Participants were scheduled at months 6 and 12 to repeat HIV testing (among HIV‐negative participants) or to review their treatment history (among HIV‐positive participants).

### 2.6 Retention strategies

All Offline group participants received an appointment slip and telephone/text reminder a few times prior to the next scheduled date. The Mixed and Online group participants had automated appointment reminder emails and two separate reminders – one week and two days before the appointment through Facebook inbox messages, LINE chat and mobile text messages. They could also access an online platform, previously described 11, which allowed them to self‐manage their appointment date and time and communicate any questions or concerns with study staff in between clinic visits.

### 2.7 Data collection and questionnaires

A self‐administered questionnaire was used at baseline visit and every six months thereafter to collect sociodemographic, risk behaviour, social network use characteristics, HIV knowledge and experiences around stigma and discrimination.

### 2.8 Statistical analysis

For this study, a sample size of 200 in each group was calculated to give a power at 80% with a two‐sided significance level of 5% to detect a difference in the proportion of first‐time testers of ≥12.5% between the Offline (assuming to be 40% to 60%) and the Mixed/Online groups.

Data were reported overall and by self‐selected groups and by gender as frequency and proportion for categorical parameters and median (interquartile range, IQR) for continuous parameters.

Predictors of poor study retention (defined as missing month 12 visit, regardless of being able to be contacted) and unsuccessful ART initiation (defined as failure to initiate ART during the study period) were analysed using a modified Poisson Regression model to estimate relative risk (RR) for the association 12. We included the following factors, hypothesized to be associated with poor study retention and/or unsuccessful ART initiation, into the models: HIV testing preference and experience (self‐selected study group, ever been tested for HIV), sociodemographic factors (gender, age, education, occupation, income), social media use (frequency of social media use, online seeking of sexual partners), risk behaviours (age at first sex, illegal drug use including amphetamine‐type stimulants ‐ ATS, number of sexual partners, group sex, self‐perceived HIV risk), HIV knowledge (HIV acquisition and HIV prevention knowledge scores) and stigma and discrimination (gender identity disclosure, having experienced rejection due to gender identity, having experienced sexual or physical abuse). Factors with *p* < 0.20 in the univariate analyses were initially considered in the multivariable Poisson regression. Four variable selection methods were used to identify the optimal set of predictor variables for the multivariable model: entering all variables, forward selection, backward elimination and stepwise selection. The model fit was assessed by Akaike Information Criterion score. Variance Inflation Factors were used to detect collinearity among factors.

For self‐perceived HIV risk, participants were asked to rate their perceived HIV risk level as no, minimal, moderate or high, with no definition of risk level provided to them. HIV acquisition and HIV prevention knowledge scores were calculated by number of correct answers (out of 9 questions for HIV acquisition and 8 questions for HIV prevention). Median score was used as a cut‐off point to generate knowledge score variables for the model.

## 3 Results

**Figure 1:**
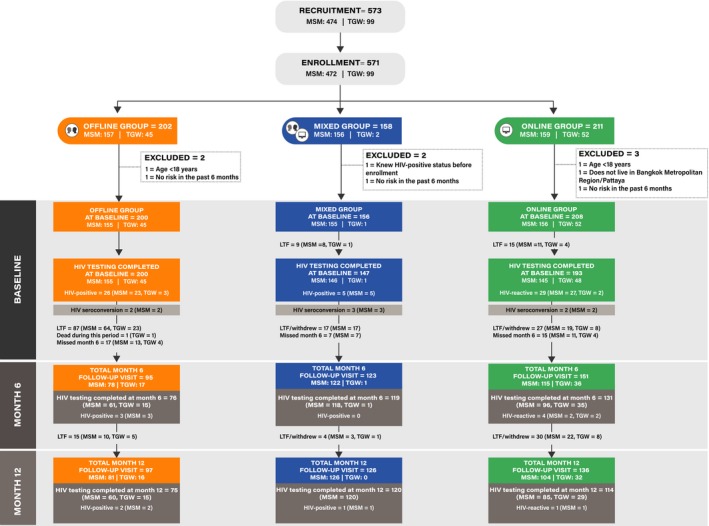
Flow of recruitment, enrolment and follow‐up of men who have sex with men (MSM) and transgender women (TGW) participants.

A total of 573 individuals (474 MSM and 99 TGW) were recruited. Of 503 individuals recruited online, 148 (39.4%) chose the Offline group, 158 (31.4%) Mixed group and 197 (39.2%) Online group. Among 70 recruited offline, 56 (80%) selected the Offline group and 14 (20%) the Online group. We enrolled 571 participants and seven were subsequently excluded (3 without risk in the past six months, 2 aged <18 years old, 1 lived outside of study area and 1 known HIV‐positive case) 9. Of 564 participants (465 MSM and 99 TGW) who contributed data to the current analysis, 200 selected the Offline group, 156 chose the Mixed group and 208 preferred the Online group (Figure 1). Of the Offline and Online group participants, 67.5% and 81.7% were enrolled by CBO staff respectively. Median (IQR) age was 26.4 (22.6 to 31.7) years. In the past six months, 68.3% had multiple sex partners, 77.4% inconsistently used condoms, 6.2% used ATS and 18.2% engaged in group sex (Table 1).

**Table 1:** Baseline characteristics of participants by self‐selected study groups

Characteristics	Overall (N = 564)	Offline group (N = 200)	Mixed group (N = 156)	Online group (N = 208)
1. Demographic data				
Gender				
MSM	465 (82.4)	155 (77.5)	154 (98.7)	156 (75)
TGW	99 (17.6)	45 (22.5)	2 (1.3)	52 (25)
Age at enrolment (years)				
Median (IQR)	26.4 (22.6 to 31.7)	25.7 (22 to 32.6)	27.5 (23.8 to 32.2)	26.2 (22.5 to 30.5)
Education				
Less than bachelor’s degree	221 (47.3)	118 (65.2)	36 (26.1)	67 (45.3)
Bachelor’s degree/above	246 (52.7)	63 (34.8)	102 (73.9)	81 (54.7)
Main occupation				
Unemployed/student	121 (26)	40 (22.1)	35 (25.5)	46 (31.3)
Employed	294 (63.2)	109 (60.2)	98 (71.5)	87 (59.2)
Service worker	50 (10.8)	32 (17.7)	4 (2.9)	14 (9.5)
Monthly income				
<458.09 USD	171 (37.6)	84 (48.3)	39 (28.5)	48 (33.3)
458.09 to 916.14 USD	192 (42.2)	66 (37.9)	59 (43.1)	67 (46.5)
916.17 to 1526.92 USD	59 (13)	14 (8)	24 (17.5)	21 (14.6)
≥1526.95 USD	33 (7.3)	10 (5.7)	15 (10.9)	8 (5.6)
2. Social media use				
Do you always use social media?				
Yes	437 (95)	160 (90.9)	134 (97.8)	143 (97.3)
No	23 (5)	16 (9.1)	3 (2.2)	4 (2.7)
How long do you spend time on social media daily? (excluding playing game)				
Weekday				
Less than 2 hours	36 (7.8)	17 (9.4)	12 (8.8)	7 (4.8)
2 to 4 hours	141 (30.5)	63 (35)	43 (31.4)	35 (24)
4 to 8 hours	195 (42.1)	75 (41.7)	43 (31.4)	77 (52.7)
8 to 24 hour	91 (19.7)	25 (13.9)	39 (28.5)	27 (18.5)
Weekend				
Less than 2 hours	25 (5.5)	11 (6.5)	7 (5.1)	7 (4.8)
2 to 4 hours	111 (24.4)	45 (26.5)	34 (24.8)	32 (21.8)
4 to 8 hours	212 (46.7)	77 (45.3)	59 (43.1)	76 (51.7)
8 to 24 hours	106 (23.3)	37 (21.8)	37 (27)	32 (21.8)
Have you ever sought sexual partner on social media?				
Yes	415 (88.9)	158 (87.3)	128 (92.8)	129 (87.2)
No	52 (11.1)	23 (12.7)	10 (7.2)	19 (12.8)
If yes, which social media have you used for seeking sexual partner? (can select more than one choice, N = 415)				
Facebook	236/415 (56.9)	109/158 (69)	56/128 (43.8)	71/129 (55)
Applications, e.g. Grindr, Jack’D, Hornet	285/415 (68.7)	84/158 (53.2)	113/128 (88.3)	88/129 (68.2)
Camfrog	92/415 (22.2)	38/158 (24.1)	18/128 (14.1)	36/129 (27.9)
Instagram	41/415 (9.9)	23/158 (14.6)	7/128 (5.5)	11/129 (8.5)
Others	10/415 (2.4)	10/158 (6.3)	0/128 (0)	0/129 (0)
What device(s) do you use for social media? (can select more than one choice)				
Mobile phone	417/470 (88.7)	159/184 (86.4)	126/138 (91.3)	132/148 (89.2)
Tablet	86/470 (18.3)	26/184 (14.1)	34/138 (24.6)	26/148 (17.6)
Personal computer (PC)	109/470 (23.2)	50/184 (27.2)	35/138 (25.4)	24/148 (16.2)
Notebook/Laptop	148/470 (31.5)	43/184 (23.4)	53/138 (38.4)	52/148 (35.1)
3. Risk behaviours				
Age at first sex (years)				
Median (IQR)	17 (15 to 19)	16 (15 to 18)	18 (16 to 19)	18 (16 to 20)
Self‐perceived HIV risk in the past six months				
No	50 (10.4)	21 (11.6)	7 (4.9)	22 (13.8)
Minimal	183 (37.9)	67 (37)	57 (40.1)	59 (36.9)
Moderate	179 (37.1)	63 (34.8)	58 (40.8)	58 (36.3)
High	71 (14.7)	30 (16.6)	20 (14.1)	21 (13.1)
Number of sexual partners in the past six months				
Had no sex	21 (4.5)	16 (8.7)	1 (0.7)	4 (2.7)
Single partner	93 (19.8)	35 (19)	28 (20.3)	30 (20.3)
Multiple partners	321 (68.3)	101 (54.9)	109 (79)	111 (75)
Unknown number of partners	35 (7.4)	32 (17.4)	0 (0)	3 (2)
Median (IQR) number of sexual partners in the past six months (N = 414)	4 (2 to 6)	3.5 (1 to 6.5)	3 (2 to 5)	4 (2 to 10)
Condom use in the past six months				
Never	46 (9.8)	27 (15.6)	10 (7.1)	9 (5.8)
Sometime	317 (67.6)	110 (63.6)	99 (70.7)	108 (69.2)
Always	106 (22.6)	36 (20.8)	31 (22.1)	39 (25)
Illegal drug use in the past six months				
Yes	166 (35.3)	66 (35.9)	50 (36.2)	50 (33.8)
No	304 (64.7)	118 (64.1)	88 (63.8)	98 (66.2)
Amphetamine‐type stimulants use in the past six months	29 (6.2)	15 (8.2)	9 (6.5)	5 (3.4)
Had group sex in the past six months				
Yes	88 (18.2)	27 (14.9)	29 (20.4)	32 (19.9)
No	396 (81.8)	154 (85.1)	113 (79.6)	129 (80.1)
4. HIV testing experience and knowledge				
Have you ever been tested for HIV before participating in the study?				
Yes	296 (63.2)	106 (57.6)	113 (81.9)	77 (52.7)
No	172 (36.8)	78 (42.4)	25 (18.1)	69 (47.3)
What is/are the barrier(s) to HIV testing? (can select more than one choice)				
Inconvenience in travelling to get the service	149 (31.8)	55 (29.9)	47 (34.1)	47 (32.2)
Afraid of getting HIV‐positive result	144 (30.8)	56 (30.4)	42 (30.4)	46 (31.5)
Inconvenient service hours	140 (29.9)	36 (19.6)	54 (39.1)	50 (34.2)
Concern about confidentiality of HIV result	112 (23.9)	41 (22.3)	32 (23.2)	39 (26.7)
Afraid of meeting people you may know	97 (20.7)	32 (17.4)	25 (18.1)	40 (27.4)
Never think about HIV testing before	67 (14.3)	32 (17.4)	17 (12.3)	18 (12.3)
Unfriendly staffs	51 (10.9)	23 (12.5)	18 (13)	10 (6.8)
Unattractive/not beautiful place	23 (4.9)	13 (7.1)	4 (2.9)	6 (4.1)
Do you want to confirm HIV status and/or link to ART if tested reactive/positive? (N = 426)				
Yes	426 (98.4)	149 (96.1)	133 (100)	144 (99.3)
No	7 (1.6)	6 (3.9)	0 (0)	1 (0.7)
If yes, please specify the time you prefer to link to ART				
Immediately	362 (87)	125 (88)	129 (97)	108 (76.6)
Within 1 month	41 (9.9)	15 (10.6)	4 (3)	22 (15.6)
1 to 3 months	8 (1.9)	1 (0.7)	0 (0)	7 (5)
3 to 6 months	5 (1.2)	1 (0.7)	0 (0)	4 (2.8)
HIV acquisition knowledge score (total score = 9 points)				
Median (IQR)	8 (7 to 9)	8 (6.5 to 9)	9 (8 to 9)	8 (7 to 9)
HIV prevention knowledge score (total score = 8 points)				
Median (IQR)	5 (4 to 6)	4 (3 to 6)	6 (5 to 6)	5 (4 to 7)
Have you ever known someone close to you who is HIV positive?				
Yes	148 (33.9)	63 (39.4)	43 (31.9)	42 (29.6)
No	234 (53.5)	77 (48.1)	78 (57.8)	79 (55.6)
Not sure/Don't know	55 (12.6)	20 (12.5)	14 (10.4)	21 (14.8)
5. Stigma and discrimination				
Family disclosure of gender identity				
Yes, self disclosure	228 (47.6)	97 (53.3)	65 (46.1)	66 (42.3)
Yes, non‐self disclosure	128 (26.7)	33 (18.1)	41 (29.1)	54 (34.6)
No	123 (25.7)	52 (28.6)	35 (24.8)	36 (23.1)
Discrimination within family due to gender identity				
No	292 (70.4)	118 (67.8)	75 (67.6)	99 (76.2)
Yes, current	9 (2.2)	2 (1.1)	6 (5.4)	1 (0.8)
Yes, past	45 (10.8)	15 (8.6)	13 (11.7)	17 (13.1)
Don’t know/not sure	69 (16.6)	39 (22.4)	17 (15.3)	13 (10)
In the past 12 months, ever been rejected from workplace due to gender identity				
Yes	43 (9)	19 (10.4)	7 (5)	17 (11)
No	434 (91)	164 (89.6)	133 (95)	137 (89)
Feel embarrassed due to gender identity				
Yes, definitely	9 (1.9)	2 (1.1)	3 (2.1)	4 (2.6)
Yes, maybe	26 (5.5)	5 (2.8)	7 (5)	14 (9)
Probably not	96 (20.1)	32 (17.7)	36 (25.5)	28 (18.1)
Definitely not	346 (72.5)	142 (78.5)	95 (67.4)	109 (70.3)
In the past 12 months, ever been sexually abused				
Yes	148 (31.1)	67 (36.2)	42 (30.2)	39 (25.7)
No	328 (68.9)	118 (63.8)	97 (69.8)	113 (74.3)
In the past 12 months, ever been physically abused				
Yes	23 (4.8)	10 (5.4)	3 (2.1)	10 (6.5)
No	456 (95.2)	175 (94.6)	138 (97.9)	143 (93.5)
In the past 12 months, ever experienced stigma and discrimination in health care setting				
Denied services (N = 165)	5 (3)	3 (6.3)	1 (1.5)	1 (1.9)
Sub‐standard services (N = 167)	13 (7.8)	10 (20.8)	1 (1.5)	2 (3.7)

IQR, interquartile range; MSM, men who have sex with men; TGW, transgender women; USD, United States Dollar.

Among 172 first‐time testers, 45.3%, 14.5% and 40.1% chose Offline, Mixed and Online groups respectively. At baseline prior to knowing their HIV status, 98.4% said they wanted to have linkage to HIV testing confirmation and/or ART services if tested reactive/positive in this study, but only 87.0% wanted to do so immediately. The intention to have immediate linkage to confirmation/ART services was reported less in the Online group (76.6%) than in the Offline (88.0%) and Mixed (97.0%) groups. Rejection from workplace due to gender identity in the past 12 months was reported by 9.0% of all participants. Embarrassment due to their gender identity was reported by 11.6% of the Online group, 3.9% of the Offline group and 7.1% of the Mixed group. The Online group were also more likely to cite the fear of meeting people they may know as a barrier to having HIV testing (27.4% vs. 17.4%) and to experience discrimination within family due to gender identity (13.9% vs. 9.7%) than the Offline group. Education level, income and HIV acquisition and prevention knowledge scores were significantly higher among Mixed group participants, compared to the other two groups.

### 3.1 HIV diagnoses by self‐selected groups

All participants in the Offline group completed HIV testing process. Nine participants in the Mixed group and 11 in the Online group did not show up for HIV testing as scheduled, and four in the Online group could not complete online, supervised, HIV self‐testing process. There were 60 participants who tested reactive/positive for HIV at baseline (Figure 1): 26/200 (13.0%) in the Offline group, 5/147 (3.4%) in the Mixed group and 29/193 (15.0%) in the Online group. In addition, 18 participants seroconverted during the study: seven in the Offline group, four in the Mixed group and seven in the Online group. An overall HIV incidence was 5.2 (95% confidence interval, CI, 3.3 to 8.2) per 100 person‐years: 8.3 (95% CI 3.9 to 17.4) in the Offline group, 3.2 (95% CI 1.2 to 8.4) in the Mixed group and 5.1 (95% CI 2.5 to 10.8) per 100 person‐years in the Online group. PrEP was accessed by 20.4%, 73.9% and 10.2%, of HIV‐negative individuals in the Offline, Mixed and Online groups, respectively.

### 3.2 Study visit attendance

Overall, 68.5% and 66.7% of participants attended month 6 and month 12 study visits. The Offline group had the lowest study visit attendance both at month 6 (47.7% vs. 83.7% vs. 78.2%) and month 12 (48.7% vs. 85.7% vs. 70.8%), compared to the Mixed and Online groups.

### 3.3 Linkage to referred facilities for HIV confirmatory testing and/or ART initiation

**Figure 2:**
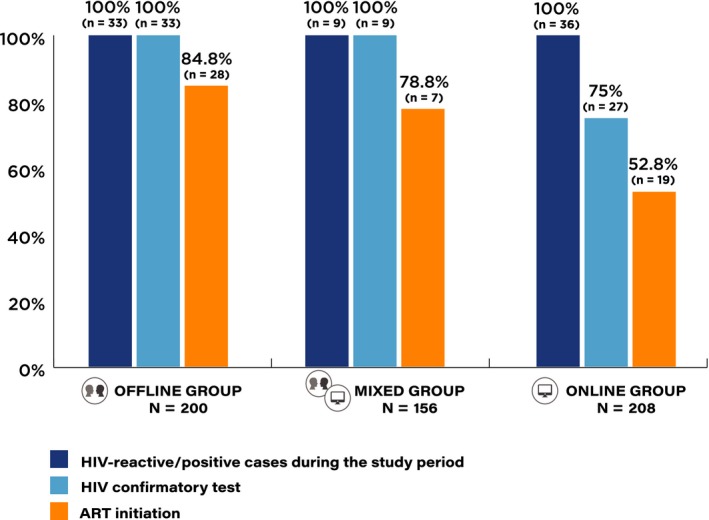
HIV testing and linkage to antiretroviral treatment (ART) cascade among men who have sex with men (MSM) and transgender women (TGW), by self‐selected study groups.

Of 78 participants who tested reactive/positive for HIV in the study, 63 (80.8%) were successfully linked to referred facilities (Figure 2): 29/33 (87.9%) in the Offline group, 7/9 (77.8%) in the Mixed group and 27/36 (75.0%) in the Online group. Among 27 Online group participants with reactive test from HIV self‐testing who visited referred facilities, 100% had their HIV‐positive status confirmed.

ART initiation was successful in 54 (78.3%) of 69 participants who were confirmed to be HIV positive (Figure 2): 28/33 (84.8%) in the Offline group, 7/9 (77.8%) in the Mixed group and 19/27 (70.4%) in the Online group. Considering all 78 participants who tested reactive/positive for HIV, overall successful ART initiation was 69.2%: lower in the Online group (52.8%) than the Offline (84.8%) and Mixed (77.8%) groups.

### 3.4 Factors associated with ‘poor study retention’

Being in the Offline group (adjusted RR 2.65, 95% CI 1.56 to 4.5), being TGW (aRR 1.42, 95% CI 1.05 to 1.91), having less than bachelor degree (aRR 1.48, 95% CI 1.6 to 2.08) and being sex workers (aRR 1.52, 95% CI 1.02 to 2.25) increased the risk for poor study retention (Table 2).

**Table 2:** Factors associated with poor retention in the study among men who have sex with men and transgender women participants

Factors	n/N (%)	Crude RR	95% CI	Adjusted RR	95% CI
Study group^a^					
Offline group	104/200 (52.0)	3.64	2.40 to 5.53	2.65	1.56 to 4.5
Mixed group	21/147 (14.3)	Ref.		Ref.	
Online group	56/193 (29.0)	2.03	1.29 to 3.20	1.56	0.9 to 2.72
Gender^a^					
MSM	132/446 (29.6)	Ref.		Ref.	
TGW	49/94 (52.1)	1.76	1.38 to 2.24	1.42	1.05 to 1.91
Age at enrolment^a^					
Age 18 to 25 years old	89/219 (40.6)	1.42	1.12 to 1.79		
Age >25 years old	92/321 (28.7)	Ref.			
Education^a^					
Less than bachelor degree	102/219 (46.6)	2.42	1.80 to 3.24	1.48	1.06 to 2.08
Bachelor degree/above	47/244 (19.3)	Ref.		Ref.	
Main occupation^a^					
Unemployed/student	34/120 (28.3)	Ref.		Ref.	
Employed	82/291 (28.2)	0.99	0.71 to 1.40	1.13	0.79 to 1.61
Service worker	33/50 (66)	2.33	1.65 to 3.30	1.52	1.02 to 2.25
Monthly income^a^					
<458.09 USD	68/170 (40)	1.5	1.15 to 1.96		
≥458.09 USD	75/281 (26.7)	Ref.			
Do you always use social media?^a^					
No	13/23 (56.5)	1.87	1.27 to 2.75	1.35	0.94 to 1.93
Yes	131/433 (30.3)	Ref.		Ref.	
Have you ever sought sexual partner on social media?					
Yes	131/411 (31.9)	0.92	0.62 to 1.37		
No	18/52 (34.6)	Ref.			
Age at first sex^a^					
<17 years old	74/173 (42.8)	1.66	1.28 to 2.16	1.15	0.87 to 1.5
≥17 years old	73/284 (25.7)	Ref.		Ref.	
Self‐perceived HIV risk in the past six months					
No/minimal	74/230 (32.2)	Ref.			
Moderate/high	82/249 (32.9)	1.02	0.79 to 1.33		
Illegal drug use in the past six months					
No	97/300 (32.3)	Ref.			
Yes	52/166 (31.3)	0.97	0.73 to 1.28		
Amphetamine‐type stimulants use in the past six months^a^					
No	135/437 (30.9)	Ref.		Ref.	
Yes	14/29 (48.3)	1.56	1.04 to 2.34	1.36	0.95 to 1.95
Number of sexual partners in the past six months					
Single partner/Had no sex	36/110 (32.7)	1.11	0.81 to 1.52		
Multiple partners	95/321 (29.6)	Ref.			
Had group sex in the past six months^a^					
No	133/392 (33.9)	1.28	0.88 to 1.87		
Yes	23/87 (26.4)	Ref.			
Have you ever been tested for HIV before participating in the study?^a^					
No	76/169 (45)	1.7	1.32 to 2.19	1.19	0.91 to 1.55
Yes	78/295 (26.4)	Ref.		Ref.	
HIV acquisition knowledge score (total = 9 points)^a^					
<8 points (less than median score)	62/134 (46.3)	1.66	1.29 to 2.14		
8 to 9 points	92/330 (27.9)	Ref.			
HIV prevention knowledge score (total = 8 points)^a^					
<5 points (less than median score)	71/169 (42)	1.49	1.16 to 1.93		
5 to 8 points	83/295 (28.1)	Ref.			
Family disclosure of gender identity					
Yes, self disclosure	74/225 (32.9)	Ref.			
Yes, non‐self disclosure	40/127 (31.5)	0.96	0.7 to 1.32		
No	41/122 (33.6)	1.02	0.75 to 1.4		
Have you ever known someone close to you who is HIV positive?					
No	73/232 (31.5)	Ref.			
Yes	44/147 (29.9)	0.95	0.7 to 1.3		
Not sure/Don't know	17/54 (31.5)	Ref.			
In the past 12 months, ever been rejected from workplace due to gender identity					
Yes	15/42 (35.7)	1.1	0.71 to 1.68		
No	140/430 (32.6)	Ref.			
In the past 12 months, ever been sexually abused					
Yes	51/147 (34.7)	1.06	0.81 to 1.4		
No	106/325 (32.6)	Ref.			
In the past 12 months, ever been physically abused^a^					
Yes	11/23 (47.8)	1.48	0.94 to 2.31		
No	146/451 (32.4)	Ref.			

CI, confidence interval; MSM, men who have sex with men; RR, relative risk; TGW, transgender women; USD, United States Dollar.

a Variables with p < 0.20 in the univariate analyses.

### 3.5 Factors associated with ‘unsuccessful ART initiation’

Choosing to be in the Online group (aRR 3.94, 95% CI 1.07 to 14.52), sexual debut before 17 years old (aRR 3.02, 95% CI 1.15 to 7.92), ATS use in the past six months (aRR 3.6, 95% CI 1.22 to 10.64) and having no or one partner in the past sxi months (aRR 3.84, 95% CI 1.36 to 10.83) increased the possibility of unsuccessful ART initiation (Table 3).

**Table 3:** Factors associated with unsuccessful initiation of antiretroviral treatment among men who have sex with men and transgender women participants who tested HIV‐reactive/positive

Factors	n/N (%)	Crude RR	95% CI	Adjusted RR	95% CI
Study group^a^					
Offline group	5/33 (15.2)	Ref.		Ref.	
Mixed group	2/9 (22.2)	1.47	0.34 to 6.41	2.53	0.34 to 18.95
Online group	17/36 (47.2)	3.12	1.29 to 7.54	3.94	1.07 to 14.52
Gender^a^					
MSM	20/71 (28.2)	Ref.			
TGW	4/7 (57.1)	2.03	0.96 to 4.28		
Age at enrolment					
Age 18 to 25 years old	8/34 (23.5)	Ref.			
Age >25 years old	16/44 (36.4)	1.55	0.75 to 3.19		
Education					
Less than bachelor degree	7/35 (20)	Ref.			
Bachelor degree/above	5/24 (20.8)	1.04	0.37 to 2.92		
Main occupation					
Unemployed/student	2/16 (12.5)	Ref.			
Employed	8/38 (21.1)	1.68	0.40 to 7.16		
Service worker	1/4 (25)	2	0.23 to 17.25		
Monthly income					
<458.09 USD	6/26 (23.1)	1.15	0.42 to 3.17		
≥458.09 USD	6/30 (20)	Ref.			
Do you always use social media?					
No	0/7 (0)	–	–		
Yes	12/52 (23.1)	–	–		
Have you ever sought sexual partner on social media?					
Yes	11/57 (19.3)	0.77	0.13 to 4.64		
No	1/4 (25)	Ref.			
Age at first sex^a^					
<17 years old	9/27 (33.3)	1.83	0.74 to 4.54	3.02	1.15 to 7.92
≥17 years old	6/33 (18.2)	Ref.		Ref.	
Self‐perceived HIV risk in the past six months					
No/minimal	7/30 (23.3)	Ref.			
Moderate/high	10/37 (27)	1.16	0.50 to 2.69		
Illegal drug use in the past six months					
No	7/40 (17.5)	Ref.			
Yes	5/22 (22.7)	1.3	0.46 to 3.64		
Amphetamine‐type stimulants use in the past six months^a^					
No	9/56 (16.1)	Ref.		Ref.	
Yes	3/6 (50)	3.11	1.14 to 8.52	3.6	1.22 to 10.64
Number of sexual partners in the past six months^a^					
Single partner/ Had no sex	5/15 (33.3)	2	0.74 to 5.4	3.84	1.36 to 10.83
Multiple partners	7/42 (16.7)	Ref.		Ref.	
Had group sex in the past six months					
No	13/50 (26)	1.11	0.41 to 2.95		
Yes	4/17 (23.5)	Ref.			
Have you ever been tested for HIV before participating in the study?					
No	9/39 (23.1)	1	0.4 to 2.49		
Yes	6/26 (23.1)	Ref.			
HIV acquisition knowledge score (total = 9 points)					
<8 points (less than median score)	7/26 (26.9)	1.31	0.54 to 3.2		
8 to 9 points	8/39 (20.5)	Ref.			
HIV prevention knowledge score (total = 8 points)^a^					
<5 points (less than median score)	11/37 (29.7)	2.08	0.73 to 5.9		
5 to 8 points	4/28 (14.3)	Ref.			
Family disclosure of gender identity					
Yes, self disclosure	10/35 (28.6)	Ref.			
Yes, non‐self disclosure	3/18 (16.7)	0.53	0.17 to 1.71		
No	4/13 (30.8)	1.16	0.45 to 2.99		
Have you ever known someone close to you who is HIV positive?					
No	7/33 (21.2)	Ref.			
Yes	8/23 (34.8)	1.64	0.69 to 3.92		
Not sure/Don't know	0/5 (0)	–	–		
In the past 12 months, ever been rejected from workplace due to gender identity^a^					
Yes	5/7 (71.4)	3.57	1.78 to 7.16		
No	12/60 (20)	Ref.			
In the past 12 months, ever been sexually abused					
Yes	7/24 (29.2)	1.36	0.58 to 3.21		
No	9/42 (21.4)	Ref.			
In the past 12 months, ever been physically abused					
Yes	3/8 (37.5)	1.58	0.57 to 4.35		
No	14/59 (23.7)	Ref.			

CI, confidence interval; MSM, men who have sex with men; RR, relative risk; TGW, transgender women; USD, United States Dollar.

a Variables with *p* < 0.20 in the univariate analyses.

## 4 Discussion

We previously reported that the use of online, supervised, HIV self‐testing is feasible in Thailand and has potential to engage a high proportion of MSM and TGW who are first‐time testers and those with high HIV prevalence 9. In this paper, we demonstrated that MSM and TGW who chose to have online, supervised, HIV self‐testing had a high HIV incidence at 5.2 per 100 person‐years. We found that 75.0% of 36 MSM and TGW who tested reactive through online, supervised, HIV self‐testing subsequently accessed HIV confirmatory testing and 100% of them were confirmed to have HIV infection. However, only 52.8% of these MSM and TGW who first tested reactive by HIV self‐testing successfully started ART.

Gender identity and sexuality discrimination among MSM and TGW are common and have an impact on health service uptake 13, 14, 15. A survey conducted among 202 TGW in Thailand in 2014 identified that 48% of TGW never accessed health services for gender‐affirming care and 47% of those who ever received services had negative experiences with health care 15. MSM and TGW in our study who selected to receive online, supervised, HIV self‐testing were more likely to cite the fear of meeting people they may know as a barrier to getting HIV testing than those who chose offline testing. They were also more likely to experience discrimination within family due to gender identity than those who opted for offline testing. While almost all of them stated prior to knowing their HIV status that they would like to have confirmation and link to ART if they tested reactive by HIV self‐testing, only three quarters of them said they wanted to do so immediately.

It has been very challenging to link our online, supervised, HIV self‐testing participants to conventional HIV treatment facilities once they tested reactive. Of the 27 participants (75% of those who tested reactive by HIV self‐testing) who had confirmatory test results, 20 of them had the results confirmed at the CBOs by the counsellors who had provided online, supervised, HIV self‐testing to them and two had the results confirmed by a mobile testing van sent out from a CBO to discreetly meet with them. ART initiation, however, could not happen in the CBOs. Therefore, only 19 (70.4% of those with confirmed HIV‐positive status, 52.8% of all tested reactive by HIV self‐testing) successfully started ART. Higher ART initiation rates were demonstrated among general and key populations including female sex workers and MSM through variations of community‐based ART initiation such as mobile unit, home‐based and drop‐in centre platforms, compared to standard facility‐based ART provision 16, 17, 18. A study in Vietnam also demonstrated that linkages to HIV confirmatory testing and ART among MSM self‐testers, with support from MSM lay providers, could be as high as 91% and 90% respectively 5. These findings indicated that decentralization of ART service to non‐conventional service delivery sites, such as a KP‐led CBO where non‐judgemental services including HIV testing, PrEP and PEP are delivered by lay providers who are familiar with lifestyles and needs of these subgroups of KP, has high potential to support the roll‐out of HIV self‐testing 8, 19, 20, 21. PrEP uptake among Online Group participants was comparable to previously reported uptake among MSM and TGW in Thailand 19. More than two‐thirds of our participants in the Offline and Online groups received services at the CBOs. This supported LINKAGES Thailand Project data which showed that, in 2018 alone, KP lay providers contributed to 55% of HIV testing services and 36% of new HIV diagnoses among MSM and TGW in Thailand. In addition, by the end of 2018, 55% of all Thai PrEP users accessed PrEP through these KP lay providers. As a result, Thailand has now legalized, institutionalized and started domestic financing of KPLHS to ensure its sustainability as an integrated part of health service delivery system.

ART was successfully initiated in 78.3% of the overall MSM and TGW who had confirmed HIV‐positive status in this study which was higher than 70% reported at Thailand national level by the end of 2018 22. However, this level of ART coverage would not bring the country near the ending HIV epidemic goal. More intensive and concerted efforts are needed in order to initiate ART immediately among people newly diagnosed and to initiate/re‐initiate ART for those with known status who are out of care 23, 24. Recent same‐day ART programmes have demonstrated dramatic increase in ART initiation, as well as retention and viral load suppression, among clients who had their ART started as soon as possible after their first engagement in care, with simplified clinical algorithm which does not need availability of comprehensive laboratory results 25, 26, 27, 28, 29.

In terms of study retention, we found that MSM and TGW in the Offline group who were not offered an online tool to self‐manage their appointments and communicate any concerns with study staff in between scheduled study visits did more poorly than those who were offered. Online self‐management represents a promising strategy for preventing and treating chronic conditions, such as diabetes and mental health disorders, as individuals are empowered to actively identify challenges and solve problems associated with their health conditions 30, 31, 32. Many ongoing studies are exploring the online self‐management concept for HIV testing, PrEP and ART use among MSM and TGW 33, 34, 35. Use of online social networks was reported as always by 95% of our participants, with Facebook (92%) and LINE application (91%) being the two most common networks, and 89% accessed these social networks through mobile phone 9. Integrating health self‐management tools into freely available and widely‐used online platforms, although vary by countries and region, would seem to be more feasible and sustainable than investing in new standalone applications which could risk limited access by potential users 36.

Sex workers often had to travel out of town or country and risked poor retention 37. Having virtual access to services may help to periodically serve them during certain period of life. TGW in our study also risked poor retention. Integrated hormone and sexual health services delivered by the Tangerine Clinic for TGW in Bangkok have proved to efficiently retain TGW for repeat HIV and syphilis testing, as well as increase the uptake of PrEP, as the services simultaneously address their primary health concerns 38. The model is being replicated by a few countries in the region 39.

Our study had several limitations. The number of clients tested HIV‐reactive by self‐testing in our study was quite small and hence could limit our ability to identify predictors of unsuccessful linkage to ART initiation. We could not make a claim about effectiveness of each testing approach due to the fact that this was not a randomized study with small sample size and large probability of confounding by choices of testing modality. We could not track access to HIV testing services outside of the study and therefore could underestimate seroconversion rate. We, however, were able to cross‐check with the National ART Program database and did not identify any HIV‐reactive/positive participants who were not linked to care but accessed care outside of the study. Lastly, we assessed only the use of blood‐based HIV self‐testing and all HIV self‐testing conducted were accompanied by real‐time online supervision. Therefore, results may not be generalizable to other programmes using oral fluid HIV self‐testing or non‐supervised HIV self‐testing.

## 5 Conclusions

Diverse approaches to HIV testing for people with various testing experience and risk perception are needed to maximize its public health benefit 9, 40. To end HIV by 2030, Thailand needs to urgently adopt and scale‐up various HIV testing service delivery models. HIV self‐testing delivered through online supervision is proved to be feasible in allowing Thai MSM and TGW, who may otherwise not accessing HIV services, to know their HIV status. However, linkages to HIV confirmatory testing and ART initiation once tested HIV‐reactive, as well as to HIV prevention services among HIV‐negative individuals, are key challenges. Alternative options to bring acceptable and accessible HIV test confirmation, prevention and ART services to these individuals after HIV self‐testing are needed.

## Competing interests

All authors declare no competing interest.

## Authors’ contributions

NP and PP designed the study, edited and finalized the manuscript for submission. NP also led the study and wrote the first draft of the report. NP and RM designed the analysis. RM analysed the data. JJ, LH and TS coordinated the study. PM oversaw data management. SP, PeP, ST, DM, SS, SH, NU, SJ and TJ implemented the study at their sites. SB and TP supervised laboratory procedures. All authors critically reviewed and approved the manuscript.
